# Cardiac magnetic resonance findings in neuronal ceroid lipofuscinosis: A case report

**DOI:** 10.3389/fneur.2022.942667

**Published:** 2022-08-22

**Authors:** Giancarlo Todiere, Stefania Della Vecchia, Maria Aurora Morales, Andrea Barison, Ivana Ricca, Alessandra Tessa, Elisa Colombi, Filippo Maria Santorelli

**Affiliations:** ^1^Cardiothoracic Department, Fondazione Monasterio, Pisa, Italy; ^2^Molecular Medicine, IRCCS Stella Maris, Pisa, Italy; ^3^Cardiology Department, CNR-IFC, Pisa, Italy; ^4^Child Neuropsychiatric Unit, ASL CN2 Alba-Bra, Alba, Italy

**Keywords:** neuronal ceroid lipofuscinosis, batten disease, CLN3, cardiac pathology, cardiac magnetic resonance

## Abstract

Cardiac magnetic resonance imaging (MRI) is an essential tool for the study of hypertrophic cardiomyopathies (HCM) and for differentiating HCM from conditions with increased ventricular wall thickness, such as cardiac storage diseases. Although cardiac MRI is already used for the diagnosis and characterization of some forms of storage diseases involving the myocardium, it has not yet been used to study myocardial involvement in neuronal ceroid lipofuscinosis (NCL). Here, we describe comprehensive cardiac MRI findings in a patient with the CLN3 form of NCL showing basal inferior interventricular septal hypertrophy with maintained indexed LV mass within reference values and low T1-native values. MRI findings support a finding of abnormal storage material within the myocardium in CLN3 disease. We recommend the possible routine use of cardiac MRI for early diagnosis of cardiac involvement in CLN3 disease (also termed juvenile NCL) and to monitor the effects of emerging CLN3 therapies on the myocardium as well.

## Introduction

Cardiac magnetic resonance imaging (MRI) is an essential tool for studying hypertrophic cardiomyopathies (HCM) and to differentiate HCM from conditions presenting with an increase in ventricular wall thickness, such as cardiac deposit diseases (1). Late gadolinium enhancement (LGE) represents the standard for non-invasive imaging of replacement fibrosis (2). Additionally, native T1 mapping without contrast administration improves our diagnostic power also in subjects with contraindications to gadolinium (3). Briefly, high values of native T1 can be obtained in the presence of myocardial fibrosis and edema (4), while low values can be a consequence of the accumulation of iron or fat (4). Cardiac MRI is also used in the diagnosis of Anderson-Fabry disease (AFD) cardiomyopathy, a lysosomal storage disease (LSD) characterized by intracellular accumulation of glycosphingolipids (5). Cardiac involvement in AFD includes left ventricle (LV) hypertrophy, valvular thickening, and conduction disturbances, followed by heart failure due to myocardial fibrosis induced by glycosphingolipid accumulation (6). Typical LGE pattern suggesting that AFD diagnosis is the late myocardial enhancement localized at the infero-postero-lateral region with the unaffected endocardium. Furthermore, another finding is a low myocardial non-contrast T1 that could be used as a marker to detect myocardial glycosphingolipid storage (7).

Because of these properties, the role of cardiac MRI can be justified for tissue characterization of other forms of storage diseases involving the myocardium. Neuronal ceroid lipofuscinoses (NCL) is a group of autosomal recessive forms of LSD that can present with a combination of epilepsy, psychomotor decline, dementia, and blindness (8). Interestingly, CLN3/juvenile NCL can be complicated by cardiac involvement in the late stages (9). Anecdotal cases reported changes in myocardial function and storage material accumulation in cardiomyocytes of CLN3 patients, among the commonest NCL (10). A study involving 29 CLN3 children reported progressive cardiac involvement with repolarization disturbances at ECG, ventricular hypertrophy, and sinus node dysfunction, and showed an association between inverted T waves and increased risk of death (11). Results from another study involving 42 CLN3 patients evaluated every 6 months by ECG and echocardiography reported hypertrophy and bradycardia as the most common cardiac abnormalities (9). Histopathological analyses on human biopsies showed cytoplasm vacuolization and auto-fluorescent storage material in the cardiomyocytes, especially in the conduction system (12–14) with fibrosis (13), fatty infiltration (13), and calcifications (12). Electron microscopy showed curvilinear bodies, fingerprint patterns, and lipofuscin deposits in cardiomyocytes (15). Here, we describe for the first-time cardiac involvement in a patient with CLN3 disease focusing on features observed on cardiac MRI.

## Case description

### Patient

**Figure 1:**
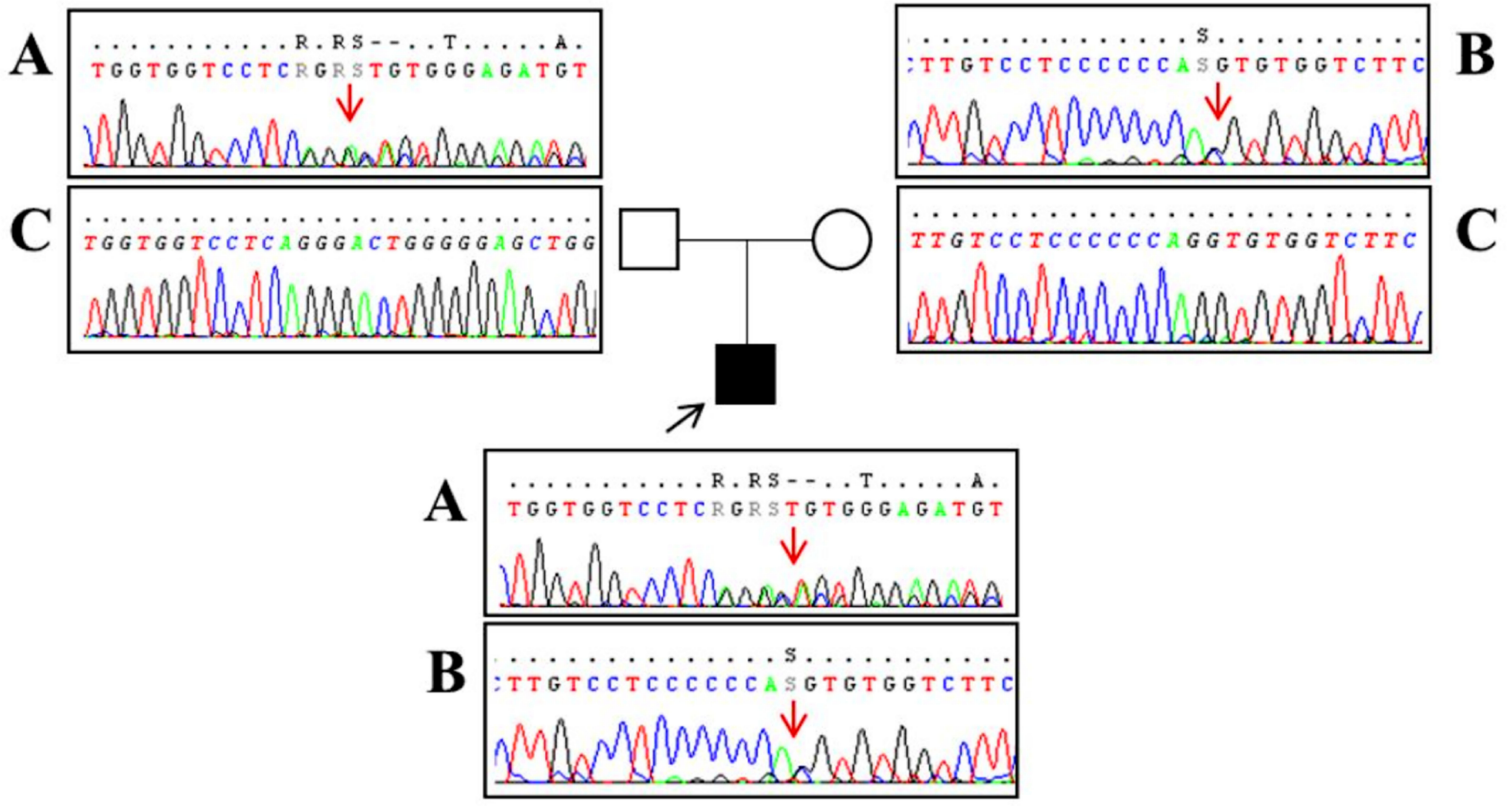
Sanger sequencing analysis performed in our patient (black square) revealed a two-base deletion (c.558_559delAG) in exon 8 of the CLN3 gene [**(A)** red arrow] and a splice-site mutation (c.461-1G>C) upstream exon 7 [**(B)** red arrow]. Segregation analysis showed that the father (empty square) carries the c.558_559delAG mutation **(A)** and a wild-type allele **(C)** whereas the mother (empty circle) has the splice-site mutation **(B)** and a wild-type allele **(C)**. Base positions are referred to as the NM_000086 reference sequence (Ensembl genome browser, https://www.ensembl.org/).

A 16-year-old boy with the juvenile phenotype of CLN3 disease underwent a contrast-enhanced cardiac MRI including a native T1 mapping assessment. Informed consent was obtained from the patient's parents. He had an unremarkable prenatal and perinatal history. He could sit unassisted at 6 months, walk unsupported at 18 months, and speak first words at 12 months. Progressive visual impairment began at 5 years with a diagnosis of retinal dystrophy. The first generalized tonic-clonic seizures appeared at 7 years. Epilepsy was partially controlled with valproate (900 mg/die). At that age, brain MRI was reportedly normal. Shortly afterward, progressive psychomotor regression, irregular sleep-wake rhythm, and behavioral disturbances started. ECG and echocardiogram performed annually from the age of 7 were always normal. DNA studies revealed biallelic mutations in *CLN3*, the c.558_559delAG inherited from the father and the c.461-1G>C on the maternal allele (Figure 1). At the latest neurological exam, the patient walked a few steps with support, presenting severe spastic tetraparesis with dystonic postures and cerebellar ataxia. We also recorded blindness, dysphagia, severe cognitive decline, and dysarthria with the production of a few poorly intelligible words. Brain MRI documented mild diffuse and symmetrical hyperintensity in T2-weighted images involving cerebral and cerebellar white matter, thinning of the corpus callosum, and of the optic chiasm, global supra- and sub-tentorial atrophy. EEG detected slow background activity and paroxysmal abnormalities involving mainly the parietal, temporal, and occipital regions bilaterally. At 16 years, ECG showed sinus rhythm with normal atrioventricular conduction, and negative T waves in DIII, V5, V6 leads, and normalized QT interval in the reference range. Two-dimensional transthoracic echocardiography detected mild LV hypertrophy (end-diastolic interventricular septum wall thickness 12 mm) and normal LV ejection fraction (EF 66%, Simpson rule).

### Cardiac MRI acquisition and analysis

**Figure 2:**
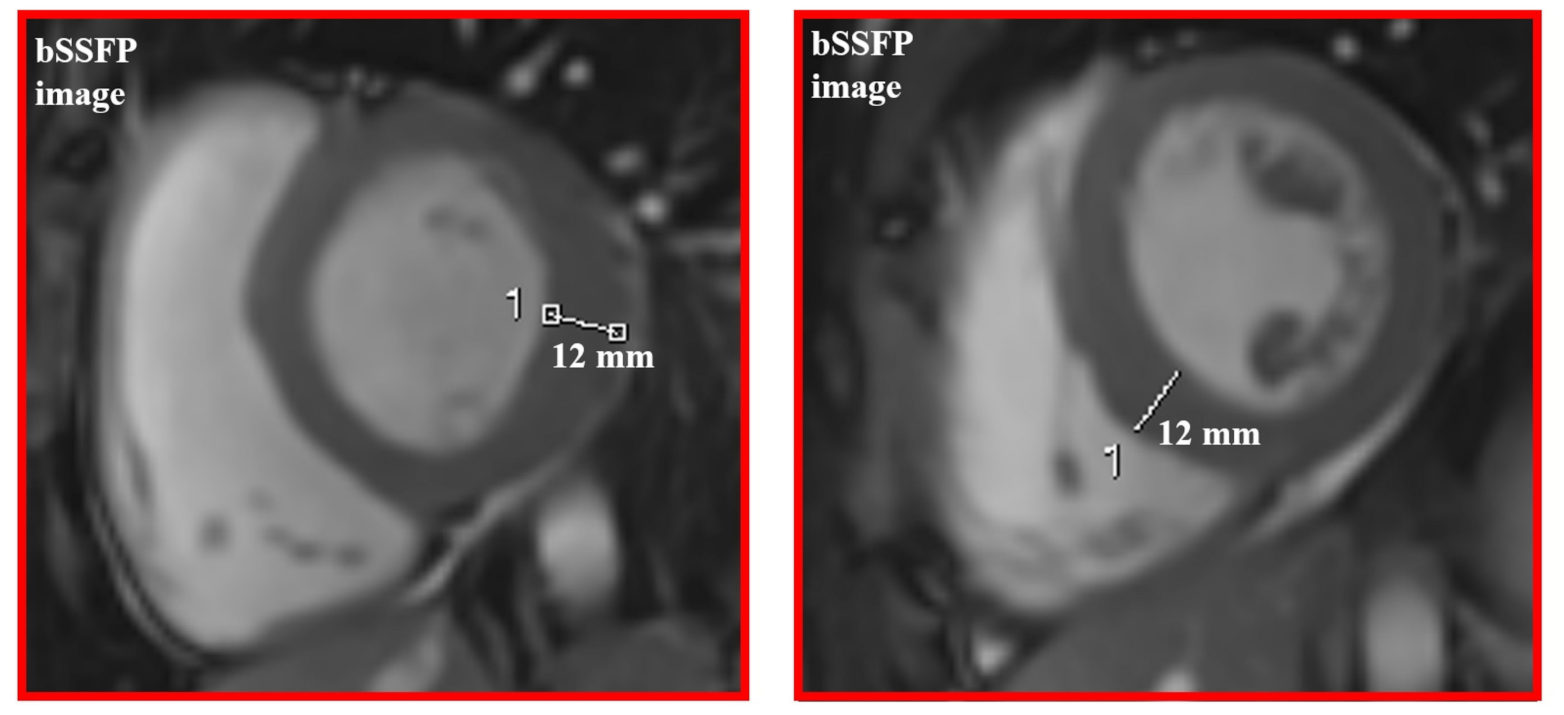
Concentric left ventricular hypertrophy of basal and mid segments on cine images.

**Figure 3:**
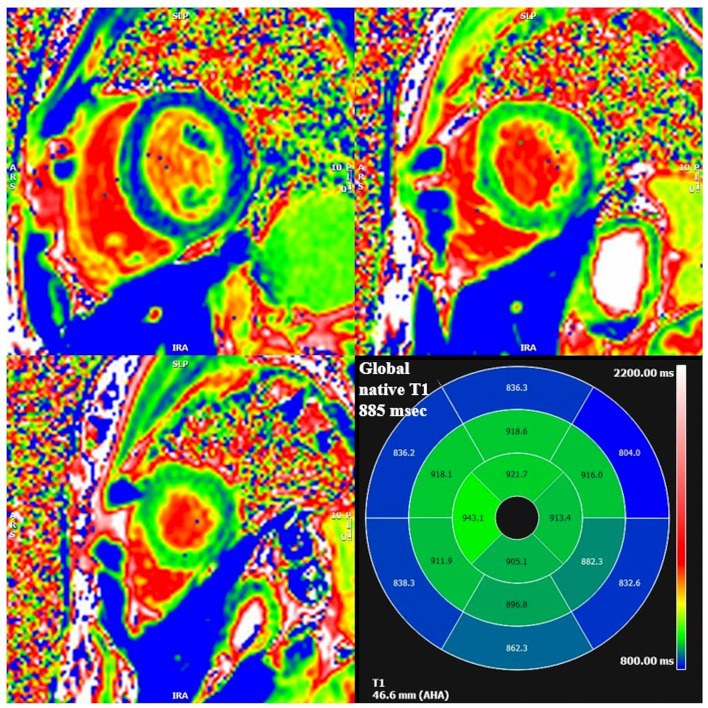
Segmental and global low native T1 mapping at MOLLI images. On the bottom right square, AHA segmentation with T1 native values of each segment. Areas of low native T1 are highlighted in blue.

**Figure 4:**
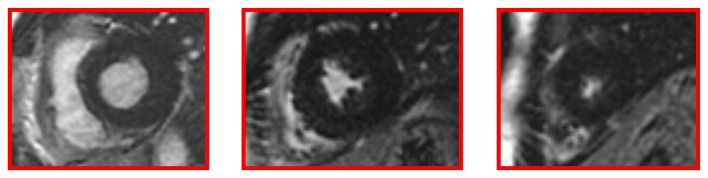
Late gadolinium enhancement (LGE) images without hyperintense myocardial areas at the left ventricle.

Cardiac MRI, using a 1.5 Tesla system (GE Healthcare SIGNA Artist) confirmed normal indexed biventricular volumes for age and BSA, and systolic function (EF 69%), with hypertrophy of basal inferior interventricular septum (12 mm) but maintaining indexed LV mass in reference values (74 g/m^2^) (Figure 2). Before the administration of the contrast agent, a sequence MOLLI SSFP for native T1 mapping was performed. Native T1 values were calculated by dedicated postprocessing software (Circle Cardiovascular Imaging, Alberta, Canada) and significantly shortened than normal values of the Laboratory were obtained (global T1 of LV was 885 ms, normal value ≥ 928 ms) (Figure 3) (16). No areas of LGE (defined as hyperintense areas after administration of gadoteric acid ≥ 6 standard deviations than a region of interest in the background) were detected in the myocardium, covering the entire left ventricle from the mitral valve plane to the apex (Figure 4).

## Discussion

We presented comprehensive cardiac MRI findings in a patient with CLN3 disease. We found basal inferior interventricular septal hypertrophy with maintained indexed LV mass within reference values and low T1-native values. Our findings in this case of CLN3 disease confirm that some types of NCL are characterized by cardiac involvement with excess storage of ceroid and lipofuscin-like materials in cardiomyocytes (12–14). As in patients with AFD, low myocardial non-contrast T1 observed in our patient could be used as a surrogate marker to detect myocardial storage also in CLN3 disease (7). While cardiac function in CLN3 patients has so far been assessed mainly by ECG and echocardiogram, the clinical use of cardiac MRI should be considered as a tool to directly and non-invasively characterize myocardial structure in the early stages of CLN3 disease, even before life-threatening events, such as advanced conduction disturbances. It is tempting to suggest the routine use of cardiac MRI for the early diagnosis of cardiac involvement in CLN3 disease and to monitor the effects of emerging therapies for CLN3 disease on the myocardium as well. However, it should be noted that a limitation in the routine use of cardiac MRI in CLN3 may be the need for sedation in pediatric patients or patients with advanced dementia.

It should be noted that our patient's genotype does not include the more frequent 1 kb founder deletion, which accounts for approximately 90% of the affected alleles in CLN3 patients (74% homozygous and 22% heterozygous) (17, 18) and leads to the formation of a protein lacking exons 7 and 8, located in the second of the four luminal loops of the protein (19). On the contrary, our patient has an unusual CLN3 genotype with a deletion (c.558_559delAG) in exon 8 and a splice-site mutation (c.461-1G > C) upstream exon 7 of the *CLN3* gene. Both mutations present in our patient have already been described; the 2bp deletion results in a frameshift at the protein level (p.Gly187Aspfs^*^48) (17), while the second is a mutation in intron 6 that is predicted to result in defective splicing (17). Many compound heterozygous mutations, including the one described here, fall within the same loop involved by 1 kb deletion, suggesting that this domain is critical for protein structure and function (17). Although milder phenotypes with slower progression have been observed, like some missense mutations (19), genotype–phenotype correlation is not well understood, neither regarding the severity of the clinical phenotype (19) nor regarding cardiac involvement. In this regard, one study established that genotype does not predict the severity of behavioral phenotype in CLN3 disease (20). Although there is a need to study cardiac MRI features even in patients with the more frequent 1 kb deletion, it is worth noting that one of the largest studies on cardiac involvement in CLN3 disease showed a high frequency of cardiac ventricular hypertrophy in their 3rd decade of life (11), a finding also confirmed in another cohort of CLN3 patients (9), suggesting that our findings are not attributable to our patient-specific mutation.

Furthermore, our patient obtained subjective benefits from the administration of Trehalose–a sugar being tested in several neurodegenerative diseases, such as NCLs (*ClinicalTrials.gov. Available online: https://clinicaltrials.gov/ct2/results?cond=trehalose&term=&cntry=&state=&city=&dist=; accessed 19 June 2022*) for its neuroprotective actions (21) and its ability to stimulate autophagy (22). In fact, an alteration of the autophagy-lysosome system has been found in many of these conditions. This is also the case with CLN3 disease. Research in various model organisms has highlighted the importance of CLN3 protein function in autophagy, indicating that it influences the expression and activity of lysosomal enzymes and modulates vesicular trafficking and autophagic degradation (23). It is not known whether Trehalose administration may also have a benefit on cardiac function. However, a new MRI study on treated patients is warranted to define any positive effects on the heart as well.

In the present study, cardiac biomarkers were not assessed when cardiac imaging was performed. Troponin and NTproBNP are suggestive of infiltration in subjects with suspected disease and normal left ventricular ejection fraction (LVEF). When cardiac abnormalities are detected by imaging techniques (either ultrasounds or cardiac MRI), their role becomes relevant during follow-up, to assess the progression of the disease and the possible effect of treatment.

Being a case report, the results are limited to a single case with an unusual genotype, thus firm conclusions cannot be drawn. Presentation of cardiac MRI results on a single patient and the absence of a baseline cardiac MRI examination are the main weak points, though the study brings original information worth exploring further. The strength is that, to our best knowledge, this is the first study of NCL-associated heart disease by cardiac MRI.

## Data availability statement

The datasets presented in this article are not readily available because of ethical and privacy restrictions. Requests to access the datasets should be directed to the corresponding author.

## Ethics statement

The studies involving human participants were reviewed and approved by the CEPR-Tuscany Region Pediatric Ethics Committee. Written informed consent to participate in this study was provided by the participants' legal guardian/next of kin. Written informed consent was obtained from the participants' legal guardian/next of kin for the publication of any potentially identifiable images or data included in this article.

## Author contributions

GT and FS conceived the experiment. GT, MM, and AB performed cardiac MR analysis. SD, MM, IR, EC, and AT collected clinical, imaging, and genetic data. AT performed the genetic analysis. GT wrote the first draft of the manuscript. All authors contributed to the article and approved the submitted version.

## Funding

This research project was funded by the Tuscany Region (Bando Ricerca Salute 2018, DEM AGING) (to FS) and the Italian Ministry of Health, Ricerca Corrente 5 × 1,000 (to AT and FS).

## Conflict of interest

The authors declare that the research was conducted in the absence of any commercial or financial relationships that could be construed as a potential conflict of interest.

## Publisher's note

All claims expressed in this article are solely those of the authors and do not necessarily represent those of their affiliated organizations, or those of the publisher, the editors and the reviewers. Any product that may be evaluated in this article, or claim that may be made by its manufacturer, is not guaranteed or endorsed by the publisher.
